# Abstracts from Foreign Journals

**Published:** 1898-12

**Authors:** J. Preston Hoskins

**Affiliations:** Princeton, N.J.


### ABSTRACTS FROM FOREIGN JOURNALS.

### GERMAN.

UNDER THE DIRECTION OF J. PRESTON HOSKINS, PH.D.,
PRINCETON, N. J.

ANEMONE NEMOROSA (grove anemone), according to the investigations of Prof. G. Müller and his assistant, C. Krause, is not a poisonous plant. This is contrary to the view of Haubner, Dammann, Fröhner, Arnold, Tereg, and Robert. The investigations of Prof. Müller were made upon a horse, a cow, two goats, three dogs, and several rabbits. In no case did anemone nemorosa cause any disturbance in digestion, diarrhœa, or diminish the appetite. The principal effect consists in an irritation of the kidneys, which, however, does not lead to inflammation, but only increases the quantity of urine, which becomes specifically thinner. In the dog, after taking fresh plants or the sap which has been pressed, the urine assumes a yellowish-red coloration due not to bloody matter, but to the coloring matter of the grove anemone. The plant affects further the milk-glands, causing congestion of the same, and to a moderate degree, blood in the milk, which has the odor and taste of the plant, and is not fit for use. A distillation of the plant causes local irritation. Applied subcutaneously, it causes inflammation, but never suppuration. The increase of urine also occurs when it is thus applied, but no effect can be traced on the milk-glands. Dry plants and the roots exert no visible influence on the animal.—*Thierärztliches Centralblatt*, June 1, 1898.

TETANUS THERAPEUTICS.—Nocard, as is generally known, uses his antitetanus serum in all operations and wounds which are likely to superinduce lockjaw. Nevertheless, his serum has been used as a remedy for tetanus which had already set in. In the *Veterinary Record*, the recovery of a mule which had been seized with lockjaw, after castration, is reported. The animal received two drachms of serum after thirty-six hours, again after forty-eight hours, and on the sixth day another injection of one drachm.—*Idem.*

APPLICATION OF MEDICINE IN PULVERIZED FORM TO THE ORGANS OF RESPIRATION.—Official veterinarian Neubarth, in

Zullichau, has constructed a "duster," by means of which he applies medicine in pulverized form (usually dermatol, with a small percentage of iodoform) to the mucous membrane of the larynx and pharynx. The instrument is made of metal, and consists of a bellows or blower, a chamber for the reception of the medicine, and a tube shaped like an olive at the end, about 35 cm. long and 5 mm. thick. The tube is shoved along the base of the nostril into the nose. The instrument can be had from Haubner, in Berlin. Price, 9.75 marks.—*Idem.*

Tenacity of the Aphtha-contagion.—In connection with the already-published observations of Gotteswinter in regard to the contagiousness of offal which had lain seven months in the dung-pit, District Veterinarian Strebel publishes the following in the *Schweizer Archiv.* He, as well as many other Swiss veterinarians, had in the years 1872–1873 the opportunity to observe that a pretty large number of infected cattle, among them some that had not entirely recovered from the first attack, fell sick again with the aphtha-contagion within six to ten weeks. He and others confirm the statement that the same animals fell sick with the same contagion three different times in the same year. These facts show that the recovery once from the hoof- and mouth-disease gives the animal only little or no immunity at all against new attacks of this disease. Dr. Strebel had frequent opportunity to observe the tenacity of the virus of aphtha-contagion in the Alps. As soon as an infected herd had left the pastures the floors of the cow-sheds were customarily well cleansed, but not disinfected, and afterward the doors were left open, so that the stalls were thoroughly ventilated and the excrement and secretive materials which by chance still remained might be thoroughly dried out. If such stables were occupied before the end of three weeks with new cattle—young cattle—in a majority of cases they fell sick, never, however, if under the circumstances described, the stables were not occupied until after the end of a month. The thorough drying out of excrement pregnant with virus is therefore a good means of killing the aphtha-contagion virus. In the usual stables of the lower lands the virus can remain active for a long period where there is no or only superficial disinfection.—*Idem.*

Infection of Man with the Hoof- and Mouth-disease.—An attendant of the slaughter-house in Dresden was infected with hoof- and mouth-disease, while caring for an infected herd, in the following manner: He was smoking at his work and touched his cigar with his dirty hands. He was afterward seized with a violent fever, and blisters appeared in his mouth and on his toes. The man was unable to work for a week. In the neighborhood of Zabern, further, two milkmaids were similarly afflicted after they had drunk from a milk-bucket the milk of cows which had not yet fully recovered from the aphtha-contagion.—*Idem.*

Etiology of Nettlerash in Swine.—Friedberger and Fröhner, Nocard and Leclainche distinguish nettlerash or nettle fever from erysipelas and swine-pest, characterizing it as an independent disease. For Leblanc, on the other hand, it has no pathological entity of its own. This condition, in his view, is certainly a symptomatic manifestation of erysipelas, perhaps of swine-pest. Jensen, also, is of the same opinion, and regards nettlerash as a weakened form of erysipelas; Konze also, is of the same opinion, and he has observed both diseases at the same time. Prietsch, in a herd of swine infected with erysipelas, observed symptoms of nettlerash. Ries has published a very interesting observation supporting Leblanc's view. In a hog which had died of subcutaneous erysipelas he found, on postmortem examination, very marked symptoms of inflammation on the valve of the heart. Four weeks before the same hog had been sick with nettlerash. The second hog, after it had likewise shown symptoms of nettlerash, continued to walk unsteadily and died seven weeks after the appearance of the eruption. The autopsy showed lesions of the mitral—endocarditis.—*Idem.*

Trichosis on the Tongue of Cattle (by Dr. Anacker).—The bases of the tongue mass is often inflammatorily irritated by the food which is only imperfectly chewed at the first mastication. At this point it is almost smooth in the middle, thinner than in other places, and somewhat flatly depressed; it is the point of least resistance. The mucous membrane becomes inflamed, infiltrated, and finally ulcerous. The ulcer is round, depressed, with its periphery swollen and hardened. Out of it spring whitish-yellow or brownish hair from ½ to 1 cm. in length, more or less crowded together or standing in bundles; at times the hairs are found in a transverse ulcerous split in the mucous membrane. That it is here a question of real hairs has been proved microscopically by the author. The whole process is accompanied often, by suffering, shown in chewing and biting. According to common report, this pain decreases when the hairs are cut off, and increases again with their growth. If trichosis of the tongue interferes with eating, it is of importance for the veterinarian, and he should never neglect to take out the tongue and examine it carefully. A cautious, slow taking up of hard foods, frequent changing of the food bolus in the mouth, spitting and a final dropping of the bolus, or refusal to take hard food are symptoms which point to the existence of trichosis. Healing is difficult, and can be brought about best by caustics. Anacker recommends: Argentum nitrate in solid form, concentrated solutions of acids, *e.g.*, acid hydrochlor., ac. nitric, ac. chromic and ac. picric. During this treatment with caustics only liquid and soft food should be given, such as bran mashes and boiled carrots. Green hay should not be given until healing is nearly completed. The ulcer on the tongue is to be kept clean and disinfected until it heals. A sharp spoon-shaped instrument is well adapted for scaling out the ulcer and removing the hairs. During this operation a local anæsthetic, such as cocaine solution, can be applied to avoid causing too much pain.—*Idem.*

To Stop the Flow of Blood.—A new means for stopping the flow of blood was recommended a short time ago to the Society of Hospitals in Paris by Paul Carnot. The same is the well-known product gelatin. In a solution of common salt enough gelatin is dissolved to make a 5 to 10 per cent. solution. It is then sterilized twice by putting into a steam-bath for a quarter of an hour. An antiseptic, such as carbol solution, can be added if desirable. At the ordinary temperature it becomes a solid mass, and must be liquefied over boiling water immediately before using. In internal hemorrhages, if the source is accessible, a small sterilized wad of cotton thoroughly saturated with gelatin can be introduced. The solution can also be squirted in with a syringe or applied upon a strip of gauze. In cases of bleeding at the nose the nasal muscles should first be cleansed with warm water. The gelatin solution can then be applied with a small syringe, and then the saturated wad of cotton can be introduced. Care must be taken not to apply the solution too hot, as its effect is greatly decreased by heat.—*Idem.*

A Contribution to the Question of Tuberculin Injection in Cattle.—At a recent meeting of the Agricultural Society in Rendsburg, Germany, Professor Ostertag, of Berlin, read a paper on this subject from which the following is taken : Slaughterhouse statistics, which twenty-five years ago showed only 3 to 5 per cent. of the cattle afflicted with tuberculosis, now show 10 to 40 per cent. This number, however, is even exceeded by those which are afforded by tuberculin injections. In large cattle herds even 50 per cent. and over have reacted. With such a spread of the disease the profits of cattle-raising must have sensibly diminished. If, for the purpose of exterminating the disease, all cattle were tested and those sold which reacted it would be a practical impossibility to carry it out. For that reason the method advocated by Bang in Copenhagen is the simplest and most practical, because it works toward the end in the most guarded manner. Bang concludes from his investigations that there can be no objection to breeding cattle which have been found to be tuberculous by the tuberculin-test because by far the greater majority of tuberculous cows produce healthy calves. The calves must be fed only on boiled milk and kept in special stalls where they do not come in contact with tuberculous animals. The breeder should not be afraid of finding a large percentage of tuberculous animals among his herds, for this is the case with every breeder who has introduced imported cattle for breeding purposes.

His fears, also, on account of the impossibility to sell the milk are ungrounded, because it has been scientifically demonstrated that only the milk of such tuberculous cows as have already grown very thin, or which suffer from tuberculosis of the udder, is injurious to health. On the other hand, the milk of cows which have merely reacted on the tuberculin test is not to be regarded as dangerous. Finally, the sale of animals that have reacted is a point at which many breeders take umbrage, since they believe that such animals can only be sold at a great loss. This is not the case, however, since tuberculous processes may exist for years in an animal without spreading perceptibly.—*Idem.*

---

THE MICROBE OF AFRICAN CATTLE PLAGUE. Dr. Kolbe, assistant to Koch, believes that the microbe which causes the South African cattle plague is so minute as to probably be beyond the visual range of the most powerful microscope. Koch's investigations of the disease, extending over nearly two years, failed to detect the presence of a specific microbe that could have caused the disease. Nevertheless, Koch claims that he can greatly lessen the ravages of the disease by the use of his protective serum.

